# Utilization of Postnatal Care Services among Thai Women during the COVID-19 Pandemic: Results of a Web-Based Survey

**DOI:** 10.3390/ijerph19116536

**Published:** 2022-05-27

**Authors:** Yin Min Aye, Soo Jung Kim, Wichukorn Suriyawongpaisal, Seo Ah Hong, Yan-Shing Chang

**Affiliations:** 1 ASEAN Institute for Health Development, Mahidol University, Nakhon Pathom 73170, Thailand; smurfyinmin123@gmail.com (Y.M.A.); wichukorn.sur@mahidol.ac.th (W.S.); 2 Department of Health Sciences, Hamburg University of Applied Sciences, 20099 Hamburg, Germany; skim615@gmail.com; 3 Florence Nightingale Faculty of Nursing, Midwifery & Palliative Care, King’s College London, London SE1 8WA, UK; yan-shing.chang@kcl.ac.uk

**Keywords:** postnatal care service, women, barriers, Thailand, COVID-19

## Abstract

The postnatal period is an underserved aspect of maternity care, potentially worsened by the COVID-19 pandemic. This study aims to identify postnatal care (PNC) use by health personnel within the 42 days of childbirth among postpartum mothers in Thailand. This web-based, cross-sectional study was conducted from July to October 2021 (*n* = 840). Multiple binary and ordinal logistic regressions were conducted to predict three outcome variables (≥2 times, ≥3 times, or level of PNC use). Women who received PNC were in low numbers (≥2: 30.7% and (≥3: 12.9%), while 54.4% of women reported no barriers to access PNC, and 31.9% reported barriers, including worries over COVID-19 infection, followed by movement restrictions imposed by the government (11.7%) and the closure of healthcare centers (10%). Women working in a self-employed capacity, living in urban areas, and undergoing a Caesarean section with no/less worry about COVID-19 infection were more likely to utilize postnatal care (≥2 or number of PNC). This study provides timely information, revealing that a relatively low percentage of postpartum women received PNC, particularly among the socially deprived group. Since the fear of COVID infection is listed as a major barrier, the provision of PNC services, including a telehealth program should be considered.

## 1. Introduction

According to the World Health Organization, the postnatal period is defined as beginning immediately after the birth of the baby and extending for up to six weeks (42 days) after birth [1]. The postnatal period is a critical window of opportunity for lifesaving intervention delivery for both the mother and newborn. Since most maternal and infant deaths occur during this period [2], the service provision of maternity care at this time, known as postnatal care (PNC), is considered essential for the mother and her newborn baby in the first 42 days of life [3]. Appropriate PNC can help in preventing maternal and child morbidity and mortality, particularly in low- and middle-income countries (LMICs) [4,5]. Nevertheless, this essential service is neglected, with more emphasis placed on antenatal and intrapartum care [6]. Moreover, the coronavirus disease (COVID-19), emerging from the Hubei Province in the People’s Republic of China in December 2019 and subsequently spreading throughout the world, has resulted in alterations to healthcare structures and processes, such as reduced maternity service provision, including PNC [7,8,9,10,11,12]. However, to our knowledge, only a few studies have examined the PNC uptake using a quantitative approach during the pandemic in Ethiopia [13], Bangladesh [14], and the UK [15].

In Thailand, since the first COVID-19 case was reported on 13 January 2020 [16], a series of public health and social measures, such as wearing masks, an evening curfew, lockdown measures, social distancing, and travel restrictions were implemented under the direct leadership of the Prime Minister [16]. Thailand was considered to be the only middle-income country nominated in the global top 10 of pandemic preparedness according to the Global Health Security Health Index [17]. Despite the successful control of COVID-19 in 2020, resurgent outbreaks caused by the Alpha and Delta variants (third and fourth waves) from April to December 2021 and the Omicron variant (fifth wave) occurring since December 2021 have hit the entire country, accounting for more than 4,046,953 cases and 27,006 deaths as of 18 April 2022 [18]. A shortage of medical equipment and supplies was reported in many hospitals due to the exponential rise in cases. 

With 99.1% of births assisted by skilled health personnel, the maternal mortality ratio (MMR) in Thailand (24.6 per 100,000 live births in 2015) is much lower than the sustainable development goal (SDG) of 70 per 100,000 live births [19]. However, it is still considerably higher than the national target (15 per 100,000 live births), with significant regional variations [19]. Similar to antenatal care (ANC), PNC can be freely accessed from public hospitals through benefit packages under universal health coverage (UHC) [20]. Three postnatal contacts are recommended in three different time periods [21]. The PNC coverage was underutilized in Thailand even before the COVID-19 pandemic [21] and may have been further neglected during the pandemic. The lack of information has led to an investigation of PNC use and its barriers to access during the COVID-19 pandemic. 

Many studies have reported that the fear of COVID-19 infection hindered the uptake of maternity care services [9,10,11]. Good COVID-19 knowledge and attitude improve the overall practice of preventative measures aimed at reducing the burden of the disease. COVID-19-related knowledge, attitude, and practice (KAP) may affect the utilization of PNC. Despite some studies reporting COVID-19-related KAP among pregnant women [22,23,24], to the best of our knowledge, there is still no published study on COVID-19-related KAP and its association with PNC use among postpartum women. 

This study aims to assess the prevalence of PNC checkups, barriers to assessing PNC, and its associated factors among postpartum mothers during the COVID-19 pandemic. The findings will contribute to and assist in planning future services for women and their partners and tailoring the care to meet women’s expectations and needs as the COVID-19 pandemic continues and beyond. The findings of this study will support health professionals and policymakers by providing timely information for better utilization of postnatal maternity services to improve maternal health, leading to SDG achievement.

## 2. Materials and Methods

### 2.1. Study Design and Subjects

This is a web-based, cross-sectional study involving a total of 840 postpartum mothers in Thailand, conducted from 12 July to 28 October 2021. Data collection was performed using an online Google Form. Postpartum women aged 18–49 years old, up to six months after childbirth, with the ability to access an online survey and literate in the Thai language, completed the survey. A total of 919 women completed the survey. Those who were not living in Thailand during the survey period or did not otherwise meet the inclusion criteria were excluded. The number of respondents eligible to participate in the survey according to the inclusion criteria was 840, all of whom were included in the analysis.

Since the local government applied social distancing measures to prevent the spread of COVID-19, convenience sampling was used to recruit postpartum women. The study was advertised to midwifery and obstetric groups, and groups for mothers who were encouraged to share the study with their friends and family members, as well as online through email and social media such as Line or Facebook. Women were sent a survey link together with an online consent form, which provided information on the study aims, procedure, its voluntary nature, and confidentiality. The study was conducted according to the guidelines of the Declaration of Helsinki and approved by the Mahidol University Ethical Committee (No:2021/03-042). Mothers were informed that their participation was voluntary, and if they wanted to participate, they would need to mark the appropriate box indicating their consent, and this was then collected upon completion of the questionnaire. 

### 2.2. Variable Measures

The outcome variable, postnatal case use, was obtained by asking the question, “How many times have you received postnatal care from health personnel within 42 days of childbirth?” (response options: 1 = Never, 2 = Once, 3 = Twice, 4 = Three times, and 5 = Four times or higher). In Thailand, PNC programs involve the examination of both the mother and child. Previously, two PNC visits were recommended [25,26]. Currently, postnatal health checks on mothers and newborns are scheduled at three time points: (1) within seven days following the day of birth, (2) between 8 and 15 days after the day of birth, and (3) between 16 and 42 days after the day of birth [21]. Thus, two binary outcome variables were coded into two categories (≥2 vs. <2 times and ≥3 vs. <3 times). In addition, to assess a single-unit increase in PNC visits, an ordinal scale was employed (never, 1, 2, 3, or ≥4 visits) as an outcome variable.

The independent variables included sociodemographic factors (mother’s age, education, marital status, working status, region, residence, intended pregnancy, number of children, birth interval, mode of delivery, preterm delivery, low birth weight, and health problems during delivery and postpartum), COVID-19-related factors (family income during the COVID-19 pandemic, food insecurity changes due to COVID-19, worried about becoming infected with COVID-19, diagnosed as COVID-19 positive, ever taken a COVID-19 vaccine), and COVID-19-related KAP. The variable “family income changes during the COVID-19” was obtained by asking the open-ended question, “Currently, how much estimated income did you and/or your family earn per month during the COVID-19 in 2020 and 2021?”; the responses were categorized into three equal tertiles (low, middle, and high). The variable “food insecurity” was created from the combination of two questions—“Did you ever run out of food before the end of the month or cut down on the amount you eat to feed others in 2019 before COVID?” and “Did you ever run out of food before the end of the month or cut down on the amount you eat to feed others during COVID in 2020 and 2021?”—and then coded into three categories (still insecure over time, secure to insecure, insecure to secure/still secure over time). The variable “worried about becoming infected with COVID-19” was gained from the question “How worried/fearful are you about becoming (re)infected by the coronavirus?” Five options were provided (not at all worried, a little worried, moderately worried, very worried, and extremely worried) and then coded into three categories (not at all worried/a little worried, moderately worried, very worried/extremely worried). The variable “ever diagnosed as COVID-19 positive” was acquired from the question “Have you ever been diagnosed as COVID-19 positive?” (yes and no responses) and coded into two categories (yes, no). The variable “ever taken a COVID-19 vaccine” was attained from the question “Have you taken a COVID-19 vaccine? (response options: yes, no, and do not know)” and then coded into two categories (yes, no/do not know).

For the variable “COVID-19 knowledge”, there were questions with response options of true, false, and do not know. One point was recorded for a correct answer, with total scores ranging from 0 to 9. Attitude toward the severity and prevention of COVID-19 was assessed with seven questions, and the responses were recorded on a five-point Likert scale (“strongly disagree” to “strongly agree”), with total scores varying from 7 to 35. The practice of COVID-19 precautions was measured with six questions, and the responses were recorded on a Likert scale ranging from 1 to 4 (“never” to “always”) with total scores ranging from 6 to 24. Good knowledge, a positive attitude, and adequate practices were based on the higher score of each variable. The three variables were coded into three equal tertiles (low, moderate, or high).

### 2.3. Statistical Analyses

Statistical Package for Social Science (SPSS) version 21 (IBM Corp., Armonk, NY, USA) was used to perform the statistical analyses. The frequency and percentage of sociodemographic factors, barriers to accessing maternity care since the COVID-19 pandemic, and COVID-19-related factors, including tertiles of COVID-19 KAP and number of PNC checkups within 42 days, were used for descriptive analysis. Two binary dependent variables (≥2 vs. <2 times and ≥3 vs. <3 times) and an ordinal scale variable (never, 1, 2, 3, and ≥4) were employed in the analyses. For bivariate analyses, chi-square tests or Fisher’s exact tests were performed to assess the level of association between independent and dependent variables. Variables with a *p*-value < 0.1 in the bivariate analyses were selected for binary and ordinal logistic analysis as appropriate. Multiple logistic regression was carried out to estimate the adjusted odds ratio (AOR), with a 95% confidence interval (CI), to explore the factors associated with PNC use. A *p*-value < 0.05 was considered as significant. 

## 3. Results

### 3.1. Percentage of Postnatal Care Uptake and Barriers to Accessing Postnatal Care Services

**Figure 1:**
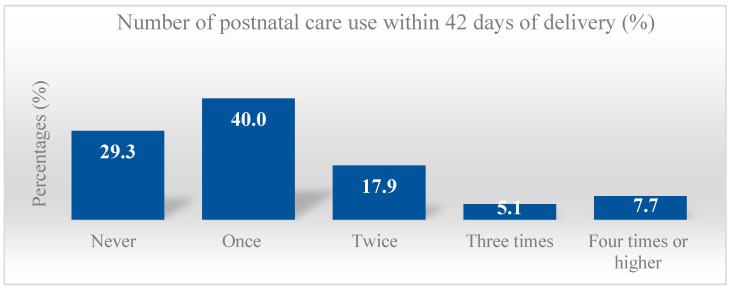
Percentage distribution of postnatal care use among postpartum women aged 18–49 years old at up to six months postpartum in Thailand during the COVID-19 pandemic.

From a total of 840 postpartum women, the percentage of women who utilized PNC was low (≥2 times 30.7% and ≥3 times 12.9%), while those receiving at least one PNC checkup was 70.7% (one time 40%, twice 17.9%, three times 5.1%, and 7.7% four times or more) (Figure 1). 

**Figure 2:**
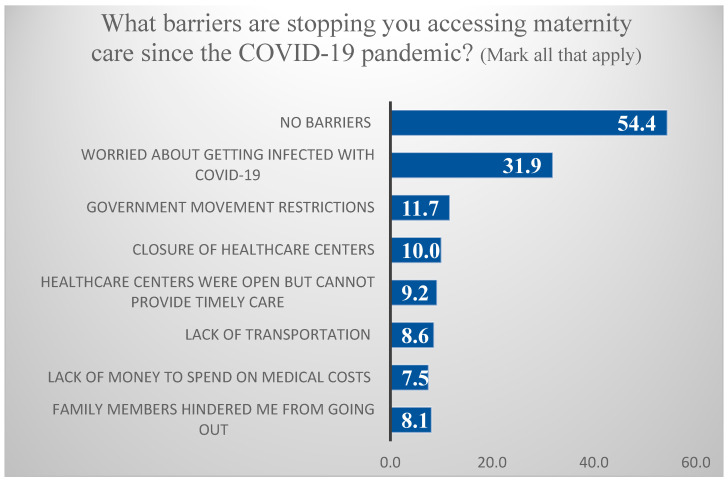
Percentage of barriers stopping the accessibility of postpartum women to maternity care during the COVID-19 pandemic. (Mark all that apply.)

As evident in Figure 2, although the majority of postpartum women (54.4%) reported having no barriers to accessing maternity care during the COVID-19 pandemic, some barriers were reported, such as being worried about becoming infected with COVID-19 (31.9%), government movement restrictions (11.7%), closure of healthcare centers (10%), healthcare centers that—although open—could not provide timely care (9.2%), lack of transportation (8.6%), lack of money to spend on medical costs (7.5%), and “family members hindered me from going out” (8.1%) during the pandemic.

### 3.2. General Characteristics of the Study Subjects

As presented in Table 1 and Table 2, the majority of participants were married (90.4%) and intended the pregnancy (83.9%). More than 50% of women were 29 years old and younger, had a secondary school and lower education level, resided in rural areas, and gave birth naturally. About 40% of women reported being extremely worried about becoming infected with COVID-19. While half of the mothers had food security during the COVID-19 pandemic, 49.5% reported food insecurity (28.1% continuous food insecurity and 21.4% experiencing food insecurity during COVID-19). Furthermore, 17% of mothers reported having been diagnosed as COVID-19 positive, while 55.8% had received at least one dose of a COVID-19 vaccine.

### 3.3. Bivariate Associations between Independent Factors and Postnatal Care Uptake

According to the bivariate analysis, independent factors such as the mother’s education, working status, residence, mode of delivery, family income during the COVID-19 pandemic, worries about COVID-19 infection, ever taken a COVID-19 vaccine, and COVID-19-related knowledge were significantly associated with the PNC uptake (≥twice and the number of uptakes) (*p* < 0.05), while urban residence, health problems during delivery, COVID-19-related knowledge, and attitude were associated with PNC visits (≥3 times) (*p* < 0.05).

### 3.4. Multivariate Association with Postnatal Care Uptake

As shown in Table 3, women who were self-employed or involved in a family-based business (AOR = 1.96, 95% CI = 1.20–3.20) had an urban residence (AOR = 1.45, 95% CI = 1.06–2.00), Cesarean section (AOR = 1.86, 95% CI = 1.34–2.57), and worried about COVID-19 infection (AOR = 1.66, 95% CI = 1.15–2.39 for no/a little worried and AOR = 1.64, 95% CI = 1.10–2.45 for moderately worried vs. very/extremely worried) were more likely to utilize PNC (≥twice). Regarding PNC visits (≥3 times), urban residence (AOR = 1.69; 95% CI = 1.12–2.57) and a COVID-19-related attitude (AOR = 1.90, 95% CI = 1.07–3.39) were associated with the uptake of these. 

Ordinal logistic analysis similarly showed that a single-unit increase in the number of PNC visits was associated with women who were self-employed or involved in a family-based business (AOR = 1.58, 95% CI = 1.04–2.40), had a Caesarean section (AOR = 1.57, 95% CI = 1.20–2.05), and worried about COVID-19 infection (AOR = 1.70, 95% CI = 1.26–2.28 for no/a little worried vs. very/extremely worried). 

## 4. Discussion

A recent study from Ethiopia reported that early PNC checkups decreased by 9.3% in the period from March to October 2020 (the first eight months of the pandemic) compared with the period from July 2019 to February 2020 [13]. Thailand still lacks nationally representative data in relation to PNC health checkups [21]. Before the pandemic, the prevalence of married women aged 15–49 years whose last living child was aged 2–11 months and who had received two postpartum checkups was 56.8% in 2020 [27] and 65.5% in 2009 [28]. Meanwhile, Thailand’s multiple indicator cluster survey (MICS) from 2015–2016 revealed that the national prevalence of PNC checkups among women aged 15–49 years with a live birth in the last two years was 22% (≥2 times) and 3% (≥3 times) [21]. Our study reveals a relatively higher percentage of PNC checkups (30.7% for ≥2 times and 12.8% for ≥3 times). On the other hand, the percentage of postpartum women who had never had a PNC checkup (29.3%) was also higher in our study than that according to the national estimate in 2015–2016 (18.2%) [21]. Although one should be cautious when interpreting the results due to the use of non-random sampling in this study, the higher percentage of women who had never used PNC in our study may be associated with COVID-19. Further studies are needed to identify the impact of COVID-19 on PNC uptake among Thai women using a nationally representative sample. Meanwhile, the discrepancy between our estimates and the national estimates regarding access to PNC checkups may be partly explained by the impact of COVID-19 mitigation measures, such as lockdowns and travel bans, and the consequent financial loss of household income during the pandemic, which may have prevented some women from utilizing healthcare services including PNC [17]. In Thailand, there are three health schemes: UHC, social security, and civil servant medical benefits, depending on which the baby’s parent is applying for. Despite there being no cost involved in accessing essential services in the public sector, some conditions, such as delivery in a private facility, or the need for extra services, such as a special room, meals, and additional drugs or treatment, may involve additional costs. Thus, financial difficulties may hinder postnatal mothers from accessing essential maternity services, resulting in socioeconomic inequality [12]. In this study, we also identified government movement restrictions (11.7%), lack of transportation (8.6%), and lack of money to spend on medical costs (7.5%) as barriers to the accessibility of PNC checkups during the pandemic. The results of the bivariate analyses in this study indicate that those with high incomes during the COVID-19 pandemic were more likely to have PNC, despite the multivariate analyses showing no significance in this respect. In Thailand, women accounted for approximately 91% of manufacturing jobs losses and 58% of jobs overall when the COVID-19 impact on the labor market was at its height during the second quarter of 2020 [29]. Socially deprived women such as employees in the informal sector, those residing in conflict-affected areas and rural communities, migrants, and minor ethnic groups, tend to suffer the most from limited movement and income reduction. Many women in the informal economy, such as market vendors, agriculture, massage therapists, domestic workers, and caregivers, face a reduction in income and unemployment and have been left with limited eligibility for social security schemes and stimulus packages by the government [30]. The findings of this study also indicated that self-employed women or those involved in a family-based business made greater use of postpartum service. The use of healthcare services depends on the ability to pay opportunity costs (time and money), as well as direct financial costs (such as medical fees, medical supplies, and transportation costs). Self-employed mothers or those involved in a family-based business are more likely to be able to pay both costs compared with housewives/unemployed or waged/employed. 

During the pandemic, some hospitals faced challenges in managing COVID-19 and protecting their health workers. Modified arrangements had to be made to allow patients access to healthcare facilities [31]. Since urban health systems in Thailand are dominated by hospital-oriented care, private clinics, and hospitals [32], women with no economic problems might seek services from private hospitals during temporary health services disruption at public hospitals during the pandemic. One study reported a lack of access to maternity care in public facilities during the pandemic, with some women attempting to seek maternity services at expensive private clinics or hospitals, which are unaffordable for the poor [33]. Although it was reported that the effect of urban–rural residence in PNC utilization was generally moderate in all Asian countries compared with sub-Saharan [34], our study revealed that those living in urban areas utilized PNC more often. People living in rural areas tend to face more obstacles in trying to access health service providers, such as longer travel distances and lack of transportation. On the other hand, those living in urban areas have more choice of healthcare providers (public hospitals and private clinics) and have easier access to the healthcare provider of their choice. Thus, it may be associated with higher PNC use in urban areas in this study. 

Meanwhile, Caesarean section delivery is positively associated with higher utilization of PNC in this study. This may be partly explained by women’s understanding that the content of postpartum service is different according to the method of delivery [20]. For example, women undergoing a Caesarean section need to take extra precautions after delivery since it takes about six weeks to recover. Thus, despite worries over COVID-19 infection, they need to go to health centers for their doctors to inspect the incision site and check the progress of recovery. Therefore, those undergoing a Caesarean section may adhere to the PNC schedule for the follow-up examination of the incision rather than participate in other postpartum examinations [26].

This study demonstrated that a third of mothers reported a fear of becoming infected with COVID-19 to be a barrier to accessing maternity care services (31.9%), and this is supported by a Thai survey [35]. Mass media mostly focuses on the casualties and adverse effects of COVID-19 on society, inducing additional stress, fear, and worry [17]. In our study, the odds of utilizing the maternal health service were found to be higher among mothers who did not fear COVID-19 infection. A study from central Ethiopia supports these findings, revealing that women who did not fear COVID-19 infection were about three times more likely to utilize maternal health services during the pandemic in comparison to those who feared infection [36]. Several studies reported that the fear of becoming infected in hospitals was the reason for lower PNC use [9,10,11,37,38,39]. 

This study showed that although the significance disappeared in the multiple logistic regression model, COVID-19-related knowledge was positively associated with PNC uptake in bivariate analyses. However, the COVID-19-related attitude remained significantly associated with PNC (≥3 times) in the multivariate model. Training on COVID-19 prevention and the provision of related information may help to enhance positive attitudes and practices toward avoiding COVID-19 infection. A study in central Ethiopia reports that mothers who practiced COVID-19 preventative measures were 5.8 times more likely to use maternal healthcare services, including postnatal care, compared with those who did not [36]. Efforts should be made to improve COVID-19-related KAP through health education among postnatal women.

This study has some limitations. Since it is a web-based, cross-sectional study, the causal relationship between independent variables and outcome cannot be established. In addition, some women residing in rural areas were illiterate and did not have a phone/computer, internet access, or a sufficient level of digital literacy to participate in this study. Furthermore, with the use of convenience sampling, women participated voluntarily in this study, and those who did not participate may have had different experiences. Therefore, caution should be applied when interpreting the results. Although this study has some limitations, it can aid the understanding of maternity service utilization by postpartum mothers in Thailand. Due to the limited information available on PNC uptake, particularly during COVID-19, the findings of this study provide timely information on PNC in Thailand, which is underutilized by mothers and health professionals, and policymakers are urged to pay special attention to this area. In particular, efforts need to be made to ensure socially deprived women living in rural areas experiencing economic problems are protected during COVID-19 and any future pandemic. While healthcare was traditionally delivered face to face during the pre-COVID-19 era, the spread of COVID-19 has accelerated the growth of new approaches to healthcare provision, such as the telehealth program, including virtual consultations or video conferencing, with the utilization of updated technologies and applications during the pandemic [40,41]. Even during the COVID-19 pandemic, face-to-face contact with health personnel was still possible through the practice of social distancing, although telephone contact tended to be the most popular form of communication. Thus, a new approach to healthcare provision, such as the telehealth program, with the utilization of updated technologies and applications, should be considered to provide maternity care services during the pandemic and beyond. A recent study revealed that the shift to telehealth during the COVID-19 pandemic generally improved patient satisfaction and reduced racial disparity, although not ideal for new patient visits or those with serious health conditions [41]. For future research, a qualitative study is recommended to provide an in-depth understanding of postnatal service utilization during the pandemic.

## 5. Conclusions

This study provides timely information, revealing that a third of participants reported never using postnatal care, and only about 13% of postpartum women accessed postnatal care three times or more during the COVID-19 pandemic. Socially deprived women made far less use of postnatal care. Since the fear of becoming infected with COVID-19 was found to be a major barrier to women’s accessibility to PNC checkups during the pandemic, the provision of postnatal care services, including the telehealth program, should be urgently considered, especially for the socially disadvantaged.

## Figures and Tables

**Table 1:** Bivariate association between sociodemographic factors and postnatal care use.

	Total		Number of Postnatal Care Uptakes											
			<2	≥2		<3	≥3		Never	1	2	3	≥4	
	*n*	(%)	%	%	*p*-Value	%	%	*p*-Value	%	%	%	%	%	*p*-Value
Total number	840	(100)	69.3	30.7		87.1	12.9		29.3	40.0	17.9	5.1	7.7	
Mother’s age (years)														
18–29	489	(58.2)	60.3	53.5	0.0645	58.6	55.6	0.5484	62.2	58.9	52.0	51.2	58.5	0.2940
30–49	351	(41.8)	39.7	46.5		41.4	44.4		37.8	41.1	48.0	48.8	41.5	
Education of mother (secondary school or lower)	458	(54.5)	58.4	45.7	0.0007	55.1	50.9	0.4212	57.3	59.2	42.0	39.6	58.5	0.0015
Marital status (married)	759	(90.4)	89.7	91.9	0.3257	90.2	91.7	0.6214	91.5	88.4	92.0	93.0	90.8	0.6160
Working status														
Waged/employed	252	(30.0)	29.9	30.2	0.0028	29.8	31.5	0.1086	28.1	31.3	29.3	27.9	33.9	0.0791
Self-employed/family business	105	(12.5)	10.0	18.2		11.7	17.6		9.8	10.1	18.7	23.3	13.9	
On maternity leave	171	(20.4)	20.1	20.9		19.9	23.1		22.8	18.2	19.3	20.9	24.6	
Housewife/unemployed	312	(37.1)	40.0	30.6		38.5	27.8		39.4	40.5	32.7	27.9	27.7	
Region														
Central (incl. Bangkok)	308	(36.7)	34.9	40.7	0.1280	36.5	38.0	0.5515	35.0	34.8	42.7	41.9	35.4	0.0685
North/Northeast	105	(12.5)	13.8	9.7		13.0	9.3		9.4	17.0	10.0	4.7	12.3	
South	427	(50.8)	51.4	49.6		50.5	52.8		55.7	48.2	47.3	53.5	52.3	
Residence (urban)	382	(45.5)	42.4	52.3	0.0079	43.6	58.3	0.0040	45.5	40.2	48.0	65.1	53.9	0.0128
Intended pregnancy (yes)	705	(83.9)	83.5	84.9	0.6158	83.6	86.1	0.5082	85.8	81.9	84.0	86.1	86.2	0.7204
Number of children (one child)	495	(58.9)	59.6	57.4	0.5400	58.3	63.0	0.9906	61.8	58.0	53.3	69.8	58.5	0.2898
Birth interval														
No sibling	436	(51.9)	52.6	50.4	0.3113	51.9	51.9	0.1330	52.4	52.7	49.3	58.1	47.7	0.3010
Less than three years	102	(12.1)	11.0	14.7		11.3	17.6		13.0	9.5	12.7	20.9	15.4	
Three years or higher	302	(36.0)	36.4	34.9		36.7	30.6		34.6	37.8	38.0	20.9	36.9	
Delivery mode (Caesarean section)	351	(41.8)	37.1	52.3	<0.0001	41.0	47.2	0.2198	35.4	38.4	56.0	32.6	56.9	<0.0001
Preterm delivery (less than 37 weeks)	173	(20.6)	21.3	19.0	0.4443	20.8	19.4	0.7514	24.8	18.8	18.7	16.3	21.5	0.3769
Low birth weight (<2.5 kg)	81	(9.6)	9.8	9.3	0.8238	9.6	10.2	0.8379	10.6	9.2	8.7	4.65	13.85	0.5543
Health problems during delivery (yes)	98	(11.7)	10.5	14.3	0.1079	10.8	17.6	0.0399	12.2	9.2	12.0	14.0	20.0	0.1572
Health problems during postpartum (yes)	73	(8.7)	8.8	8.5	0.9109	8.7	8.3	0.8878	11.0	7.1	8.7	7.0	9.2	0.5890

**Table 2:** Bivariate association between COVID-19-related factors and postnatal care use.

	Total		Number of Postnatal Care Uptakes											
			<2	≥2		<3	≥3		Never	1	2	3	≥4	
	*n*	(%)	(%)	(%)	*p*-Value	(%)	(%)	*p*-Value	(%)	(%)	(%)	(%)	(%)	*p*-Value
COVID-19-related factors														
Family income during the COVID-19 pandemic														
1st tertile (low)	278	(33.3)	34.8	29.8	0.0200	34.3	26.2	0.1200	41.1	30.2	32.4	23.8	27.7	0.0171
2nd tertile	309	(37.0)	38.4	33.7		37.0	36.4		32.9	42.4	31.8	33.3	38.5	
3rd tertile (high)	249	(29.8)	26.9	36.5		28.7	37.4		26.0	27.5	35.8	42.9	33.9	
Food insecurity before and during COVID-19														
No change (insecure–insecure)	236	(28.1)	29.2	25.6	0.2720	28.8	23.1	0.3624	29.3	29.2	27.3	20.9	24.6	0.8660
Worse (secure–insecure)	180	(21.4)	22.2	19.8		21.6	20.4		23.2	21.4	19.3	18.6	21.5	
Better or no change (secure–secure)	424	(50.5)	48.6	54.7		49.6	56.5		47.6	49.4	53.3	60.5	53.9	
Worried about COVID-19 infection														
A little worried/Not at all	299	(35.6)	34.2	38.8	0.0070	34.4	43.5	0.1452	28.5	38.4	35.3	48.8	40.0	0.0077
Moderately worried	199	(23.7)	21.6	28.3		23.8	23.1		24.8	19.4	32.0	18.6	26.2	
Very worried/Extremely worried	342	(40.7)	44.2	32.9		41.8	33.3		46.8	42.3	32.7	32.6	33.9	
Ever diagnosed as COVID-19 positive (yes)	143	(17.0)	47.0	18.2	0.5401	17.1	16.7	0.9158	18.7	14.9	19.3	11.6	20.0	0.4869
Ever taken a COVID-19 vaccine (yes)	469	(55.8)	52.9	62.4	0.0107	54.8	63.0	0.1100	50.0	55.1	62.0	74.4	55.4	0.0188
COVID-19-related KAP														
COVID-19-related knowledge														
1st tertile (low)	321	(38.2)	40.9	32.2	0.0270	39.1	32.4	0.0060	43.5	39.0	32.0	23.3	38.5	0.0117
2nd tertile	166	(19.8)	19.9	19.4		20.9	12.0		17.1	22.0	24.7	14.0	10.8	
3rd tertile (high)	353	(42.0)	39.2	48.4		40.0	55.6		39.4	39.0	43.3	62.8	50.8	
COVID-19-related attitude														
1st tertile (low)	236	(28.1)	28.4	27.5	0.3710	29.4	19.4	0.0281	28.9	28.0	33.3	16.3	21.5	0.1881
2nd tertile	364	(43.3)	44.5	40.7		43.4	42.6		46.8	42.9	39.3	41.9	43.1	
3rd tertile (high)	240	(28.6)	27.1	31.8		27.2	38.0		24.4	29.2	27.3	41.9	35.4	
COVID-19-related practice														
1st tertile (low)	275	(32.7)	35.2	27.1	0.0590	33.3	28.7	0.5590	37.4	33.6	26.0	32.6	26.2	0.4095
2nd tertile	279	(33.2)	31.4	37.2		32.7	37.0		32.1	31.0	37.3	37.2	36.9	
3rd tertile (high)	286	(34.0)	33.3	35.7		34.0	34.3		30.5	35.4	36.7	30.2	36.9	

**Table 3:** Multivariate binary and ordinal logistic regression analyses of associations between selected factors and postnatal care use.

	Binary (≥2)		Binary (≥3)		Ordinal	
	AOR	(95% CI)	AOR	(95% CI)	AOR	(95% CI)
Maternal age (18–29 vs. 30–49 years)	1.00	(0.71–1.40)				
Education of mother (secondary school or lower)	0.81	(0.56–1.16)			0.98	(0.73–1.32)
Working status						
Waged/employed vs. unemployed	1.01	(0.67–1.52)			1.06	(0.76–1.47)
Self-employed/family business vs. unemployed	1.96	(1.20–3.20)			1.58	(1.04–2.39)
On maternity leave vs. unemployed	0.88	(0.55–1.41)			0.83	(0.57–1.21)
Region						
North/Northeast vs. Central					1.09	(0.82–1.47)
South vs. Central					1.02	(0.68–1.53)
Health problems during delivery (yes vs. no)			1.67	(0.95–2.92)		
Residence (urban vs. rural)	1.45	(1.06–2.00)	1.69	(1.12–2.57)	1.21	(0.93–1.58)
Delivery mode (Caesarean section vs. vaginal)	1.86	(1.34–2.57)			1.59	(1.22–2.08)
COVID-19-related factors						
Family income during the COVID-19 pandemic						
Middle vs. Low	0.85	(0.57–1.25)			1.20	(0.87–1.64)
High vs. low	1.08	(0.70–1.69)			1.30	(0.89–1.90)
Worried about COVID-19 infection						
Not at all/A little vs. very/extremely worried	1.66	(1.15–2.39)			1.74	(1.30–2.33)
Moderately vs. very/extremely worried	1.64	(1.10–2.45)			1.32	(0.96–1.83)
Ever taken a COVID-19 vaccine (yes vs. no)	0.79	(0.56–1.10)			0.80	(0.61–1.04)
COVID-19-related KAP						
COVID-19-related knowledge						
Middle vs. Low	1.29	(0.83–2.01)	0.63	(0.32–1.23)	1.42	(1.00–2.01)
High vs. low	1.23	(0.85–1.78)	1.42	(0.89–2.25)	1.30	(0.97–1.75)
COVID-19-related attitude						
Middle vs. Low			1.35	(0.77–2.34)		
High vs. low			1.90	(1.07–3.39)		
COVID-19-related practice						
Middle vs. Low	1.30	(0.88–1.93)				
High vs. low	1.20	(0.81–1.78)				

## Data Availability

The data underlying this study cannot be made publicly available, since approval was not obtained from study participants to make their data openly available.
